# Analysis of Turbulence Effects in a Patient-Specific Aorta with Aortic Valve Stenosis

**DOI:** 10.1007/s13239-021-00536-9

**Published:** 2021-04-07

**Authors:** Emily L. Manchester, Selene Pirola, Mohammad Yousuf Salmasi, Declan P. O’Regan, Thanos Athanasiou, Xiao Yun Xu

**Affiliations:** 1 Department of Chemical Engineering, Imperial College London, South Kensington Campus, London, SW7 2AZ UK; 2 Department of Surgery and Cancer, St Mary’s Hospital, Imperial College London, London, W2 1NY UK; 3 Hammersmith Hospital, MRC London Institute of Medical Sciences Imperial College London, London, W12 0HS UK

**Keywords:** Aortic valve stenosis, Large-eddy simulation, Computational fluid dynamics, Turbulence, Kinetic energy, Wall shear stress, Energy loss

## Abstract

**Abstract:**

Blood flow in the aorta is often assumed laminar, however aortic valve pathologies may induce transition to turbulence and our understanding of turbulence effects is incomplete. The aim of the study was to provide a detailed analysis of turbulence effects in aortic valve stenosis (AVS).

**Methods:**

Large-eddy simulation (LES) of flow through a patient-specific aorta with AVS was conducted. Magnetic resonance imaging (MRI) was performed and used for geometric reconstruction and patient-specific boundary conditions. Computed velocity field was compared with 4D flow MRI to check qualitative and quantitative consistency. The effect of turbulence was evaluated in terms of fluctuating kinetic energy, turbulence-related wall shear stress (WSS) and energy loss.

**Results:**

Our analysis suggested that turbulence was induced by a combination of a high velocity jet impinging on the arterial wall and a dilated ascending aorta which provided sufficient space for turbulence to develop. Turbulent WSS contributed to 40% of the total WSS in the ascending aorta and 38% in the entire aorta. Viscous and turbulent irreversible energy losses accounted for 3.9 and 2.7% of the total stroke work, respectively.

**Conclusions:**

This study demonstrates the importance of turbulence in assessing aortic haemodynamics in a patient with AVS. Neglecting the turbulent contribution to WSS could potentially result in a significant underestimation of the total WSS. Further work is warranted to extend the analysis to more AVS cases and patients with other aortic valve diseases.

**Supplementary Information:**

The online version contains supplementary material available at 10.1007/s13239-021-00536-9.

## Introduction

Aortic valve disease is a condition in which the aortic valve exhibits limited functions due to damage or pathological degradation, primarily affecting the elderly population.^31^ Aortic valve stenosis (AVS) is a type of valve disease and is defined by a narrowing of the valve during systole such that the valve leaflets do not open fully, restricting blood flow to the aorta and affecting stroke work.^12^ Left untreated, aortic valve disease can lead to numerous secondary diseases including ascending aorta dilatation, aneurysm, atherosclerosis and left ventricular hypertrophy amongst others.^13^,^30^,^36^ It has been suggested that localised high wall shear stress (WSS) as a result of a high-velocity jet impinging on the ascending aortic wall is likely to cause progressive thinning and weakening of the aortic wall.^42^

Haemodynamics in the aorta is complex, exhibiting sophisticated interactions between primary and secondary flow features. Aortic valve diseases may add to this complexity by inducing transition to turbulence in the aorta^37^—a process that is not yet well understood. Several recent studies have estimated turbulence production in the aorta by means of 4D flow magnetic resonance imaging (MRI).^7^,^16^,^21^ Although promising, 4D flow MRI has limited spatial and temporal resolutions, compromising the accuracy of parameters derived from spatial gradients of measured velocities. On the other hand, extensive numerical studies of aortic haemodynamics have been reported, but only a few considered laminar-to-turbulence transition in the aorta,^3^,^4^,^23^,^24^,^27^,^45^ especially in the presence of aortic valve pathologies in patient-specific settings.^46^

Large-eddy simulation (LES) is a numerical method which directly resolves large scale velocity fluctuations and consequently, is capable of modelling laminar, transitional and turbulence features. LES methodologies have been successfully used in biologically relevant studies of idealised^25^,^39^ and patient-specific^22^–^24^,^45^,^46^ geometries which focused on accurately predicting flow features in transitional flows. Numerical results from our previous study of transitional flow^25^ were benchmarked against experimental laser Doppler velocimetry and particle image velocimetry measurements, demonstrating the suitability of LES in capturing challenging flow characteristics relevant to arterial haemodynamics.

Lantz *et al.*^24^ conducted LES of flow through a healthy adult aorta with a Reynolds number of up to 6500 at peak systole, where they decomposed WSS into phase-averaged and fluctuating components; allowing for the quantification of turbulent WSS. In disturbed regions they found that point-wise turbulent WSS could exceed phase-averaged WSS during part of systole, highlighting the importance in considering turbulence features. The same authors^23^ also conducted LES analysis of a patient with an aortic coarctation; pre- and post-intervention, to understand the effects of aortic coarctation on turbulence formation. They found elevated levels of turbulence throughout the systolic phase with the turbulence kinetic energy being an order of magnitude smaller than the phase-averaged kinetic energy. Miyazaki *et al.*^27^ validated pseudo patient-specific LES simulations of a healthy adult aorta and a child aorta with double aortic arch, using 4D flow MRI. The authors quantitatively compared velocities using statistical methods and assessed phase-averaged WSS and viscous energy losses, although turbulence-related parameters were not included in the study. Xu *et al.*^46^ compared LES and laminar simulations for three patient-specific aortas with dilation and different aortic valve morphologies; they found little difference in large-scale flow parameters with laminar simulations underpredicting time-averaged WSS by up to 5%. The authors observed largest differences in localised regions of highly disturbed flow—particularly in the aorta with severe aortic stenosis—although turbulence-based metrics were not quantified.

Disturbances may be present in normal and diseased aortas and our understanding of turbulence effects is incomplete, especially in the context of aortic valve diseases. This study aims to fill this gap by conducting a comprehensive analysis of flow through a patient-specific aorta with aortic valve stenosis using LES. The primary objectives are to understand the conditions under which flow transitions to turbulence, and to evaluate the effects of turbulence on kinetic energy, wall shear stress and stroke work. MRI data are used to provide patient-specific boundary conditions and for comparison with the computational results.

## Materials and Methods

### Computational Model and Numerical Method

Under pulsatile conditions flow may exhibit laminar, transitional and turbulence features all within a cardiac cycle. A numerical approach using LES is deemed most suitable due to its capability in capturing the complete range of flow states. The spatially filtered Navier–Stokes equations for an incompressible fluid are given by the filtered continuity and momentum equations

1 $$\frac{{\partial \overline{{u_{i} }} }}{{\partial x_{i} }} = 0$$

2 $$\frac{{\partial \overline{{u_{i} }} }}{\partial t} + \frac{\partial }{{\partial x_{j} }}\left( {\overline{{u }}_{i} \overline{{u }}_{j} } \right) = - \frac{1}{\rho }\frac{{\partial \bar{p}}}{{\partial x_{i} }} + \nu \frac{\partial }{{\partial x_{j} }}\left( {\frac{{\partial \overline{{u_{i} }} }}{{\partial x_{j} }}} \right) - \frac{{\partial \tau_{ij} }}{{\partial x_{j} }}$$

where $u_{i}$ is the velocity, $p$ the pressure, $\rho$ the density and $\nu$ the kinematic viscosity. An implicit LES numerically resolves the large scales of flow (denoted by the overbar) using a filter width determined by the mesh size; whilst modelling the smaller, unresolved scales using a subgrid-scale model.^35^ The subgrid-scale term $\tau_{ij}$ is given by

3 $$\tau_{ij} - \frac{1}{3}\tau_{kk} \delta_{ij} = - 2\nu_{T} \bar{S}_{ij}$$

where $\bar{S}_{ij} = \frac{1}{2}\left( {\frac{{\partial \overline{{u_{i} }} }}{{\partial x_{j} }} + \frac{{\partial \overline{{u_{j} }} }}{{\partial x_{i} }}} \right)$ is the strain-rate tensor of the resolved velocity field and $\nu_{\text{T}}$ is the modelled eddy-viscosity. We have selected the wall-adapting local eddy viscosity^29^ (WALE) model due to its proper cubic near wall behaviour

4 $$\nu_{T} = \left( {C_{W} \Delta } \right)^{2} D_{W} \left( u \right)$$

5 $$D_{\text{W}} = \frac{{\left( {S_{ij}^{d} S_{ij}^{d} } \right)^{3/2} }}{{\left( {\bar{S}_{ij} \bar{S}_{ij} } \right)^{5/2} + \left( {S_{ij}^{d} S_{ij}^{d} } \right)^{5/4} }}$$

where $C_{\text{W}} = 0.325$ is the constant model coefficient, $\Delta$ is the filter width, $D_{\text{W}}$ is an operator specific to the WALE model and $S_{ij}^{d}$ is the traceless, symmetric part of the square of the resolved velocity gradient tensor. Numerical simulations were performed using the open source finite volume software; OpenFOAM. Fluid properties are representative of blood with a density of 1060 kg/m^3^ and a dynamic viscosity of 0.0035 Pa s. Temporal discretisation was achieved using a second-order implicit backwards Euler scheme^17^ and spatial discretisation was achieved using a second-order central differencing scheme (Gauss). Simulations were converged to a normalised residual of 1*e*−5 at each time-step for velocity and pressure. The LES methodology was previously implemented and validated in a study of transitional flow in an idealised medical device and was capable of capturing the processes of laminar to turbulence transition.^25^

Vessel walls were assumed rigid with a no-slip boundary condition. A three-element Windkessel model^44^ was imposed at the three branch and descending thoracic aorta outlets. The Windkessel parameters were calculated using measured blood pressures and flow rates as described in Reference 32. A list of the parameters used in this study can be found in Supplementary Material.

### Computational Mesh

Three structured meshes; M1, M2 and M3 consisting of 3.4, 7.4 and 14.3 million cells respectively were generated; each with mean cell heights of 0.79, 0.53 and 0.43 mm. A mesh sensitivity study was conducted at peak systolic flow, where both peak values and largest velocity fluctuations are expected to occur. Mean WSS, turbulent WSS and turbulence kinetic energy (TKE) were analysed to assess mesh sensitivity, with parameters integrated over the entire simulated geometry as well as the ascending aorta where flow is most likely to be disturbed. Relative to the finest mesh M3, the coarsest mesh M1 showed a maximum difference of 10.6% across all parameters investigated, whilst mesh M2 showed a maximum difference of 2.3%. The contribution of the subgrid-scale model to the LES simulation can be quantified by taking the ratio of modelled to total TKE. In the ascending aorta, meshes M1, M2 and M3 had contributions of 7.5, 4.9 and 3.7%, indicating that the majority (at least 92.5%) of the flow field was resolved in all meshes. Mesh characteristics and a complete comparison of the parameters analysed are given in Supplementary Material. Based on the results, mesh M2 was deemed sufficiently resolved and has been used for the remainder of the study.

A time-step sensitivity analysis was then conducted on mesh M2 and three time-steps of 1*e*−3, 2*e*−4 and 1*e*−4 s were considered. The same parameters used in the mesh sensitivity study were compared and found that a time-step of 2*e*−4 s was suitable, with errors less than 1% relative to the smallest time-step. This time-step ensured a mean Courant–Friedrichs–Lewy (CFL) number less than 1 throughout the cardiac cycle.

### Data Acquisition and MR Image Processing

A patient with severe aortic valve stenosis was recruited from St Bartholomew’s Hospital (London, UK) and 4D flow MRI was performed on a Siemens 3T scanner at Hammersmith Hospital (London, UK). The study received ethical approval from the Health Research Authority and Regional Ethics Committee (17/NI/0160) and was sponsored by the Imperial College London Joint Research and Compliance Office, as defined under the sponsorship requirements of the Research Governance Framework (2005). Magnetic resonance (MR) scans were cardiac and breath gated to reduce interference effects and optimise image quality. Images were acquired in the standard aortic aligned axis. Central aortic pressure measurement was acquired using a brachial cuff connected to a purpose-built device with a specialised algorithm (Sphygmacor, AtCor Medical, Sydney, Aus).

**Figure 1:**
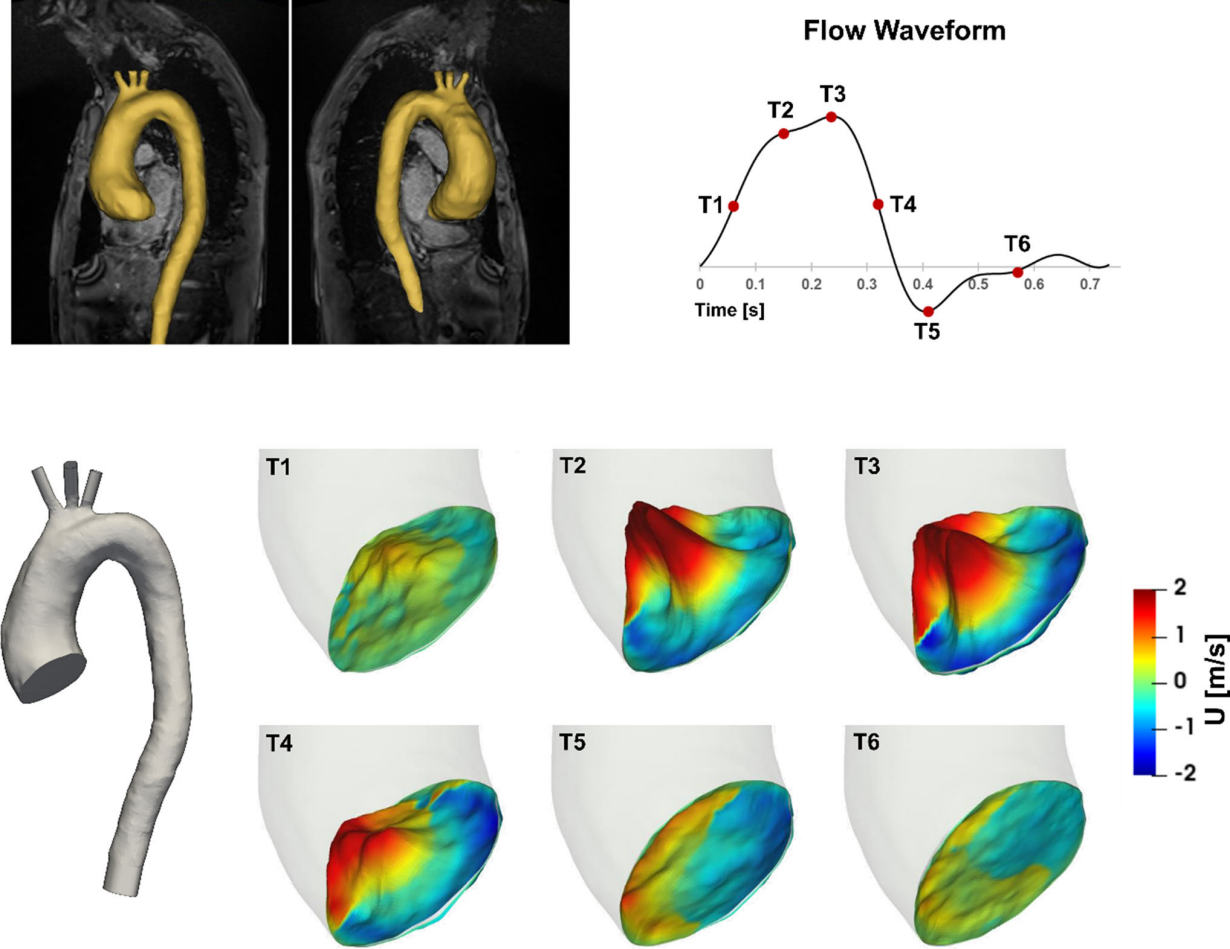
Pre-processing of the computational model. Top: 3D reconstruction of the aorta and flow waveform with key time points. Bottom: Reconstructed aorta cut at inlet and outlet planes, and 3D velocity contours at key times throughout the cardiac cycle.

MR images (voxel size 0.74 × 0.74 × 1.5 mm) were used to reconstruct the 3D patient-specific aortic geometry which included the thoracic aorta and arch branches using Materialise Mimics (v20.0, Materialise, Leuven, Belgium). The model inlet was placed in the ascending aorta, just downstream of the sinotubular junction. 4D flow MRI voxel size was 2 × 2 × 2 mm and 20 time points were reconstructed per average cardiac cycle. Anterior-posterior, foot-head and right-left velocity components were acquired with velocity encoding parameters (VENC) set to 2.2, 3 and 3 m/s, respectively. The three components of velocity were extracted from 4D flow MRI at the model inlet using an in-house MATLAB code.^33^ These values were interpolated onto the computational inlet mesh, producing 3D velocity profiles over the entire cardiac cycle representative of the patient data. The reconstructed geometry, inlet velocity contours and flow waveform are shown in Fig. 1.

### Numerical Convergence

**Figure 2:**
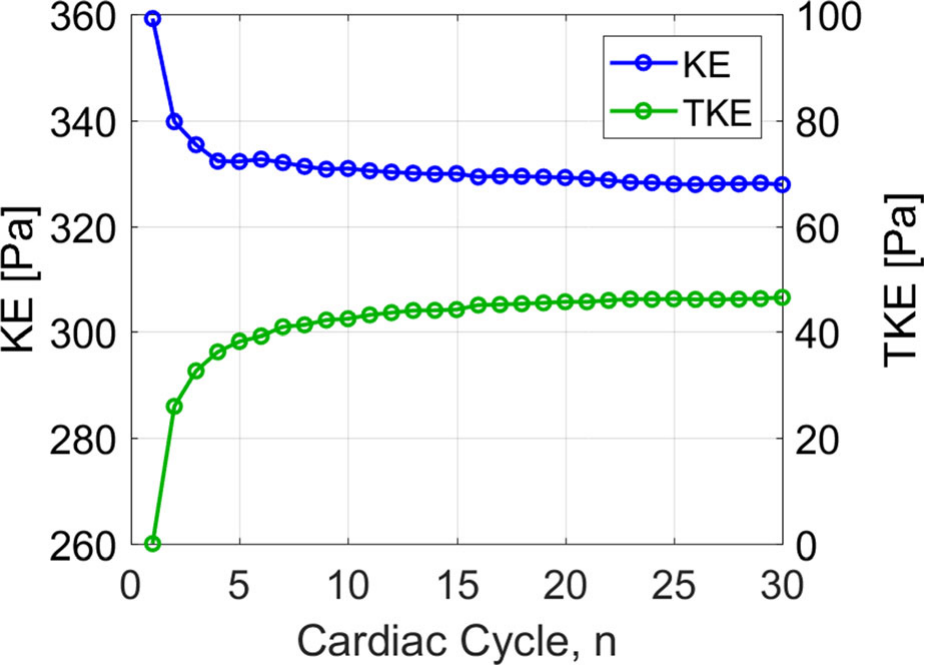
Running phase-average of mean kinetic energy (KE) and turbulence kinetic energy (TKE), integrated over the ascending aorta at peak systole.

Due to the highly unsteady nature of turbulent flows, a sufficient number of cardiac cycles must be simulated to ensure statistical convergence of turbulence parameters. The phase-average operator $\left\langle \cdot \right\rangle$ of a given variable $\phi$ is calculated as

6 $$\left\langle \phi \right\rangle \left( {\varvec{x},t} \right) = \frac{1}{N}\mathop \sum \limits_{n = 0}^{N - 1} \phi \left( {\varvec{x},t + nT} \right)$$

where *N* is the total number of cardiac cycles, *T* is the period of the cardiac cycle and t is a specified time within a cycle, e.g., peak systole. For disturbed pulsatile flows, the phase-average provides the correct representation of a variable at any given time in the cardiac cycle. Figure 2 shows the running phase-averaged kinetic energy (KE) and TKE integrated over the ascending aorta at peak systole. Relative to the phase-average over 30 cardiac cycles (*n* = 30), the KE and TKE converge to less than 1% error by the 9th and 23rd cardiac cycle, respectively. Similar analysis of the phase-averaged and turbulent wall shear stresses found errors less than 1% by the 8th and 23rd cycles, respectively. As such, 30 cardiac cycles were deemed more than sufficient for statistical convergence of both mean and turbulence parameters. Results were analysed in terms of kinetic energy, wall shear stress and energy loss, by making use of the full 30 cardiac cycles. In total 31 cardiac cycles were simulated, with the first cycle neglected in order to eliminate initialisation effects.

### Post-processing of Results

An instantaneous variable subjected to disturbances can be decomposed into phase-averaged and fluctuating components using a method similar to Reynolds decomposition

7 $$\phi \left( {\varvec{x},t} \right) = \left\langle \phi \right\rangle \left( {\varvec{x},t} \right) + \phi^{\prime}\left( {\varvec{x},t} \right)$$

where the phase-averaged component is given by Eq. (6) and the fluctuating component is defined as the root-mean-square (RMS) of the instantaneous and phase-averaged fields.

8 $$\phi^{\prime}\left( {\varvec{x},t} \right) = \sqrt {\frac{1}{N}\mathop \sum \limits_{n = 0}^{N - 1} \left( {\phi \left( {\varvec{x},t + nT} \right) - \left\langle \phi \right\rangle \left( {\varvec{x},t} \right)} \right)^{2} }$$

Note that by the definition of RMS, $\phi^{\prime}$ already represents the phase-average. The treatment of any given variable, as described in Eqs. (6–8) will form the basis for subsequent post-processing, with all parameters representing the phase-average unless stated otherwise. Kinetic energies of the phase-averaged and fluctuating velocity components are defined respectively as

9 $${\text{KE}} = \frac{\rho }{2}\mathop \sum \limits_{i} \left\langle {u_{i} } \right\rangle^{2} \left[ {\text{Pa}} \right]$$

10 $${\text{TKE}} = \frac{\rho }{2}\mathop \sum \limits_{i} u{^{\prime}}_{i}^{2} \left[ {\text{Pa}} \right]$$

where $\left\langle {u_{i} } \right\rangle$ is the phase-averaged velocity component $\left( {i = 1, 2, 3} \right)$ and $u^{\prime}_{i}$ is the fluctuating velocity component. Equations (6–8) can be applied to the instantaneous WSS, resulting in phase-averaged (laminar) WSS and fluctuating (turbulent) WSS. Integrating any of the properties over the full cardiac cycle results in a cycle-average, referred to as the time-average (e.g., time-averaged wall shear stress).

11 $$\bar{\phi }\left( {\varvec{x},t} \right) = \frac{1}{T}\mathop \int \limits_{0}^{T} \left\langle \phi \right\rangle \left( {\varvec{x},t} \right) {\text{d}}t$$

The rate of energy loss can be estimated by integrating the viscous dissipation function over the volume of the aorta.

12 $$\dot{E}_{L} = \frac{\mu }{2}\int {\mathop \sum \limits_{i,j} \left( {\frac{{\partial \left\langle {u_{i} } \right\rangle }}{{\partial x_{j} }} + \frac{{\partial \left\langle {u_{j} } \right\rangle }}{{\partial x_{i} }}} \right)^{2} {\text{d}}v \left[ W \right]}$$

where $\mu$ is the dynamic viscosity. Turbulent dissipation can similarly be calculated by replacing $\left\langle {u_{i,j} } \right\rangle$ with $u^{\prime}_{i,j}$ in Eq. (12). Integrating the viscous and turbulent dissipation over the cardiac cycle gives the net viscous and turbulent energy losses, $E_{L}$ (Joule), respectively.

All simulations were performed on 180 cores using the Cirrus UK National Tier-2 HPC Service at EPCC. Results were post-processed using Paraview.

## Results

### Comparisons Between LES Predicted and 4D Flow MRI Measured Velocities

**Figure 3:**
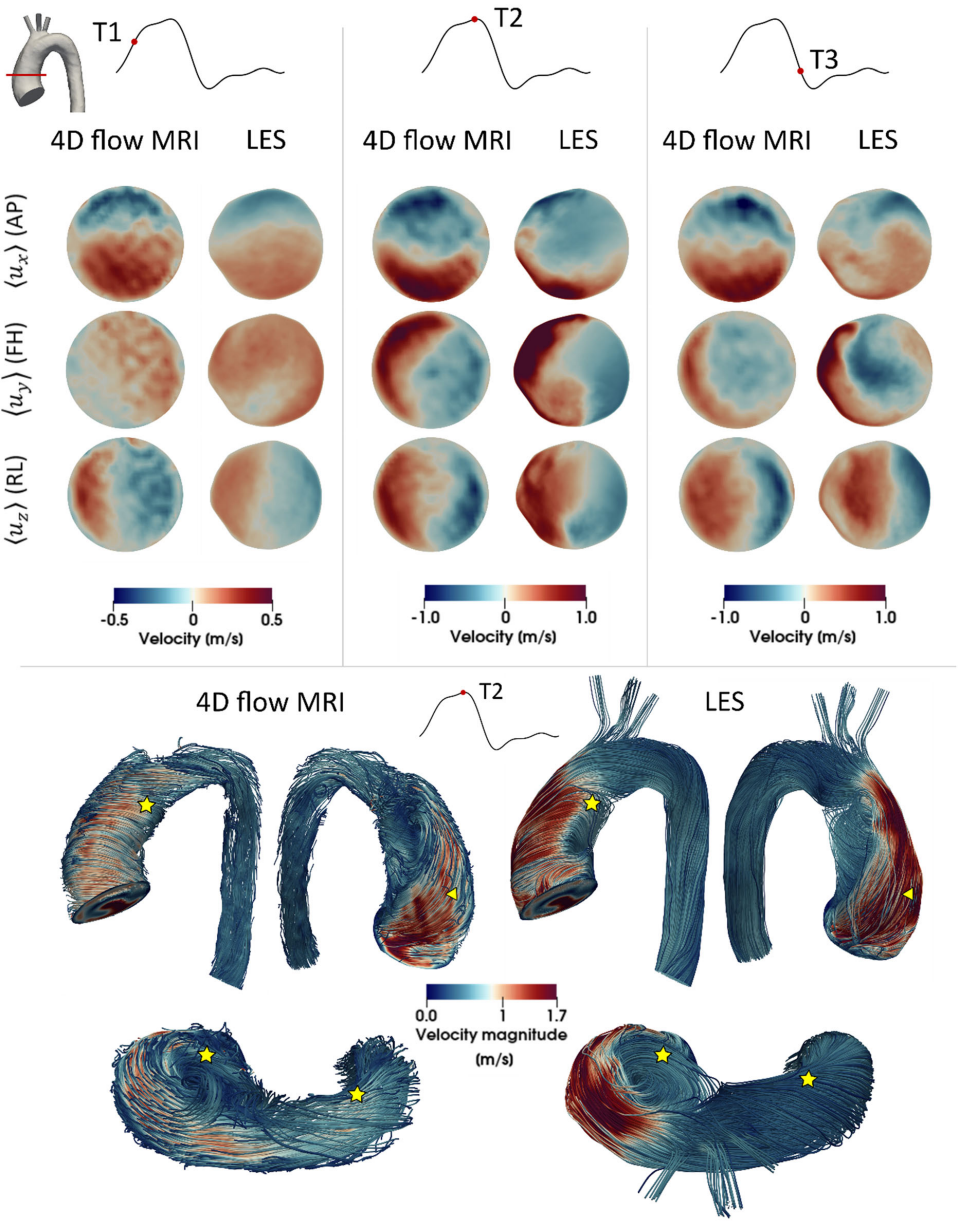
Comparison of velocity contours and streamlines between 4D flow MRI and LES results. Velocity contours (top) shown are at a plane in the ascending aorta as indicated, plotted over three time points corresponding to systolic acceleration, peak systole and systolic deceleration. The three components of velocity are anterior-posterior (AP), foot-head (FH) and right-left (RL) which correspond to *x*, *y*, *z* co-ordinates respectively. Velocity magnitude streamlines (bottom) are at peak systole. Stars and triangles denote regions of interest.

Qualitative and quantitative comparisons of velocities obtained with LES and 4D flow MRI were made to assess the reliability of the numerical results. Figure 3 shows the respective velocity contours at a plane in the ascending aorta at three time points in the systolic phase, as well as velocity magnitude streamlines at peak systole. A good qualitative agreement is observed between 4D flow MRI and LES for all velocity components at each time point. Large-scale flow features are also similar between the two data sets; these include the skewed jet from the stenosed aortic valve impinging on the anterior vessel wall (denoted by the triangle in Fig. 3) and localised regions of helical flow (denoted by the stars). Further comparisons of velocity contours in the descending aorta during the systolic phase are presented in Supplementary Material.

For quantitative comparison of the LES results and 4D flow MRI, we used the Pearson’s correlation method which gives a normalised measure of the covariance of two variables, quantifying the linearity between two datasets.^28^ This statistical approach is commonly employed in biomedical image-based research^1^,^2^ and has been used to compare 4D flow MRI measured velocities to numerical predictions and experimental data.^27^, ^34^ In this study we calculated the Pearson’s product-moment correlation coefficient (R) for the velocity field in both the entire aortic fluid domain as well as the ascending aorta where disturbed flow may occur. In general, largest differences between MRI and LES are observed at peak systole,^6^,^34^ therefore peak systole was selected for comparison. Owing to the different spatial resolutions of 4D flow MRI and LES, the LES velocity field was down sampled to the 4D flow MRI resolution allowing a pixel by pixel evaluation, as recommended in Reference 34.

**Figure 4:**
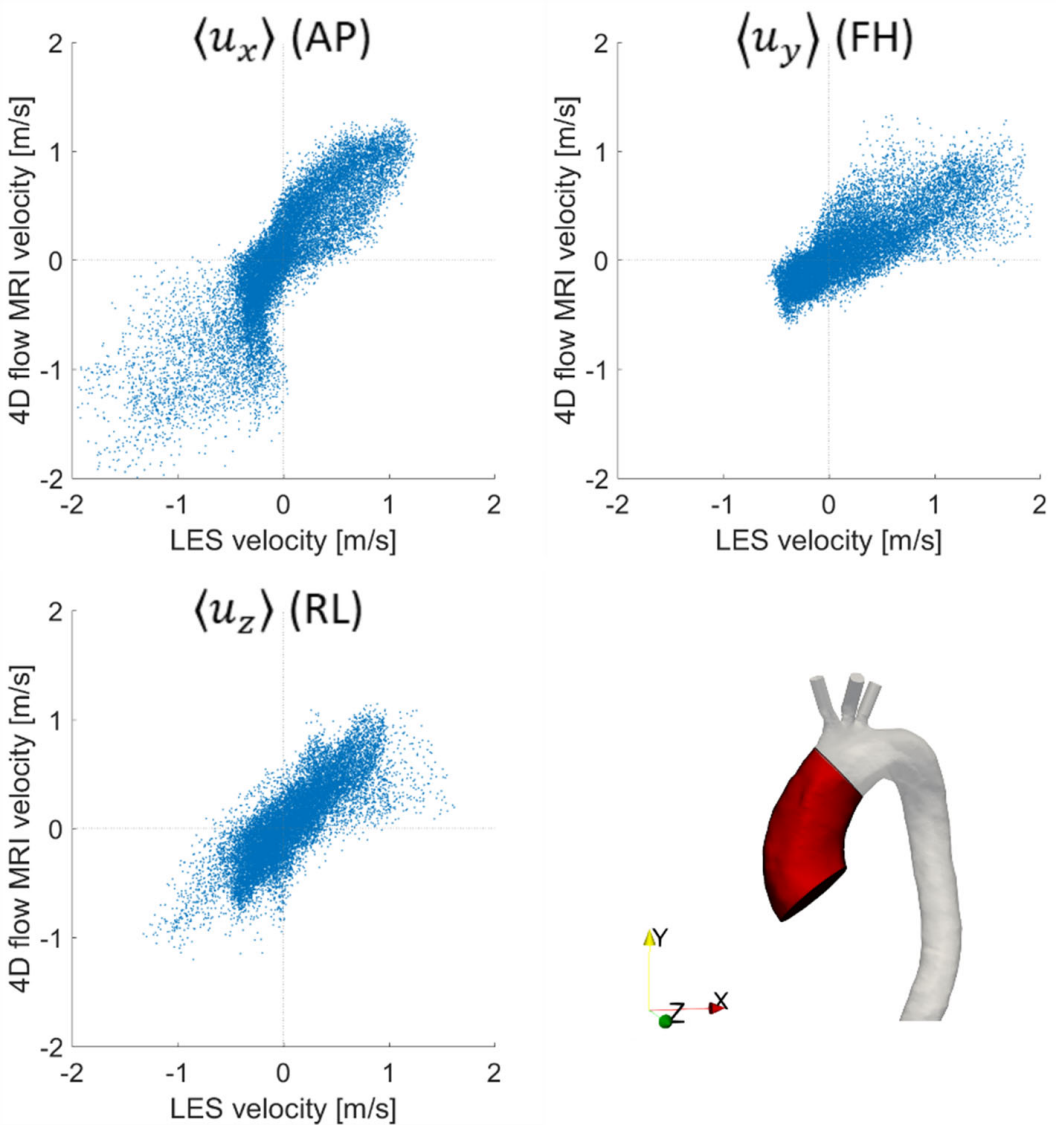
Correlation between 4D flow MRI and LES in the ascending aorta at peak systole. The ascending aorta region is highlighted in red and the phase-averaged velocity components are anterior-posterior (AP), foot-head (FH) and right-left (RL) which correspond to *x*, *y*, *z* co-ordinates respectively.

R values for the three phase-averaged components of velocity at peak systole are summarised in Table 1. An R value greater than 0.7 indicates a high positive correlation^28^ and all velocity components in this study show correlation values greater than 0.83 in the ascending aorta and greater than 0.74 in the full aorta, indicating a strong correlation between the LES predicted and MRI measured velocities. Correlation plots for velocities in the ascending aorta are shown in Fig. 4. Despite the high correlations observed, there are numerous reasons why the correlation is not even better. Low velocity regions are difficult to compare due to the signal-to-noise ratio in 4D flow MRI limiting accurate measurement of low fluid velocities. MR imaging used to reconstruct aortic geometry and 4D flow MRI were acquired at different times causing registration mismatch; effectively this means that the velocity field and aortic geometry are not perfectly aligned. Lastly, the lower spatial resolution of 4D flow MRI causes further complications including; projection errors and poor near wall resolution leading to differences between the two methods. The latter is further exacerbated by voxel averaging during image acquisition which dampens velocity gradients. The interested reader is referred to Reference 34, where these limitations are discussed thoroughly.

**Table 1:** Pearson correlation coefficients (*R*).

Region	*R*$\left( {\left\langle {u_{x} } \right\rangle } \right)$	*R*$\left( {\left\langle {u_{y} } \right\rangle } \right)$	*R*$\left( {\left\langle {u_{z} } \right\rangle } \right)$
Ascending aorta	0.87	0.83	0.85
Entire aorta	0.85	0.74	0.77

Pearson correlation coefficients (*R*) for the three components of phase-averaged velocity in the ascending aorta (top row) and entire aortic fluid domain (bottom row)

### Kinetic Energy

**Figure 5:**
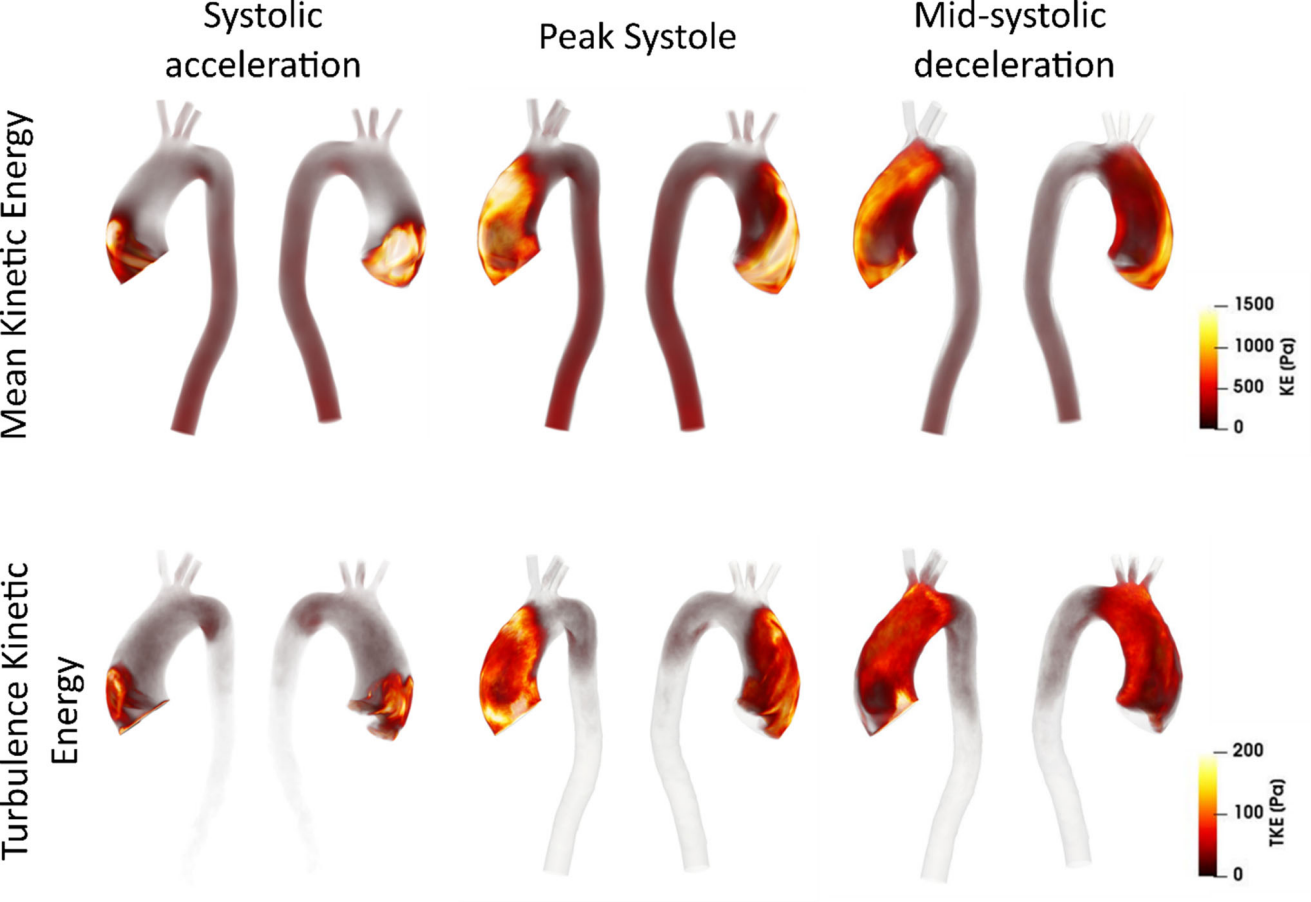
Volume rendering of mean and turbulence kinetic energies at (left to right) systolic acceleration, peak systole and mid-systolic deceleration. Note the different ranges for KE and TKE.

**Figure 6:**
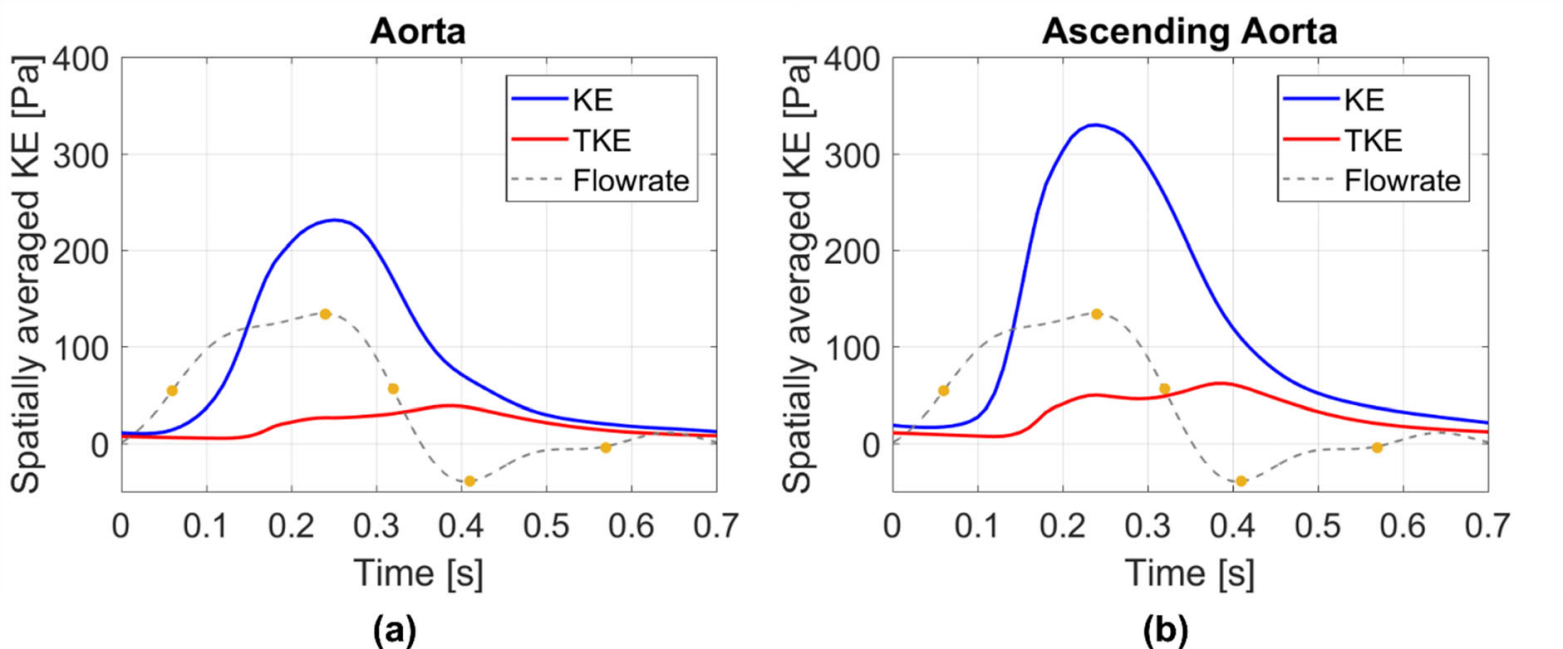
Kinetic energies spatially averaged over the entire aorta (a) and the ascending aorta (b). Plots show the phase-averaged kinetic energy (KE) and turbulent kinetic energy (TKE). Key times throughout the cardiac cycle are highlighted and refer to maximum acceleration, peak systole, maximum deceleration, end systole and mid-diastole.

Kinetic energy in a fluid domain can be decomposed into phase-averaged (KE) and turbulence (TKE) components. Phase-averaged kinetic energy is a function of the phase-averaged velocity in a fluid and as such, can be used to identify high energy regions that are associated with large scale/dominant flow features; such as high velocity blood entering the ascending aorta during systole. Turbulent kinetic energy is associated with eddies in disturbed flows and can be used to quantify the level of turbulence. TKE has been commonly used to assess turbulence effects in the aorta.^4^,^23^,^40^,^43^ Figure 5 shows volume renderings of KE (top row) and TKE (bottom row) at three time points in the cardiac cycle, and Fig. 6 shows KE and TKE spatially averaged over the entire aorta and ascending aorta.

There are high levels of KE and TKE in the ascending aorta and arch throughout the systolic phase, with peak KE an order of magnitude higher than TKE. Localised regions of high TKE generally coincide with regions of high KE, as can be seen in Fig. 5. From diastole through early systole, both KE and TKE are small and of comparable magnitudes (not shown). Spatially averaged values over the ascending aorta show a maximum average KE of 330 Pa at 0.24 s (peak systole) and a maximum average TKE of 62 Pa at 0.39 s (end systole) (Fig. 6). At end systole KE rapidly decreases whilst spatially averaged TKE peaks. It is not until diastole that turbulence steadily dissipates. Smallest values of KE and TKE are found during early systole and systolic acceleration, respectively.

### Wall Shear Stress

Wall shear stress is a measure of the shear force exerted on the arterial inner surface and has been correlated to the onset and progression of arterial diseases.^13^,^15^ In numerical simulations of biological flows, wall shear stress is typically averaged in time to represent the mean shear load over time. Similar to previous LES studies of the aorta,^3^,^24^ we decomposed WSS into phase-averaged (laminar) and fluctuating (turbulent) components to better understand both the total shear force exerted on the wall as well as the contribution from near wall disturbances.

**Figure 7:**
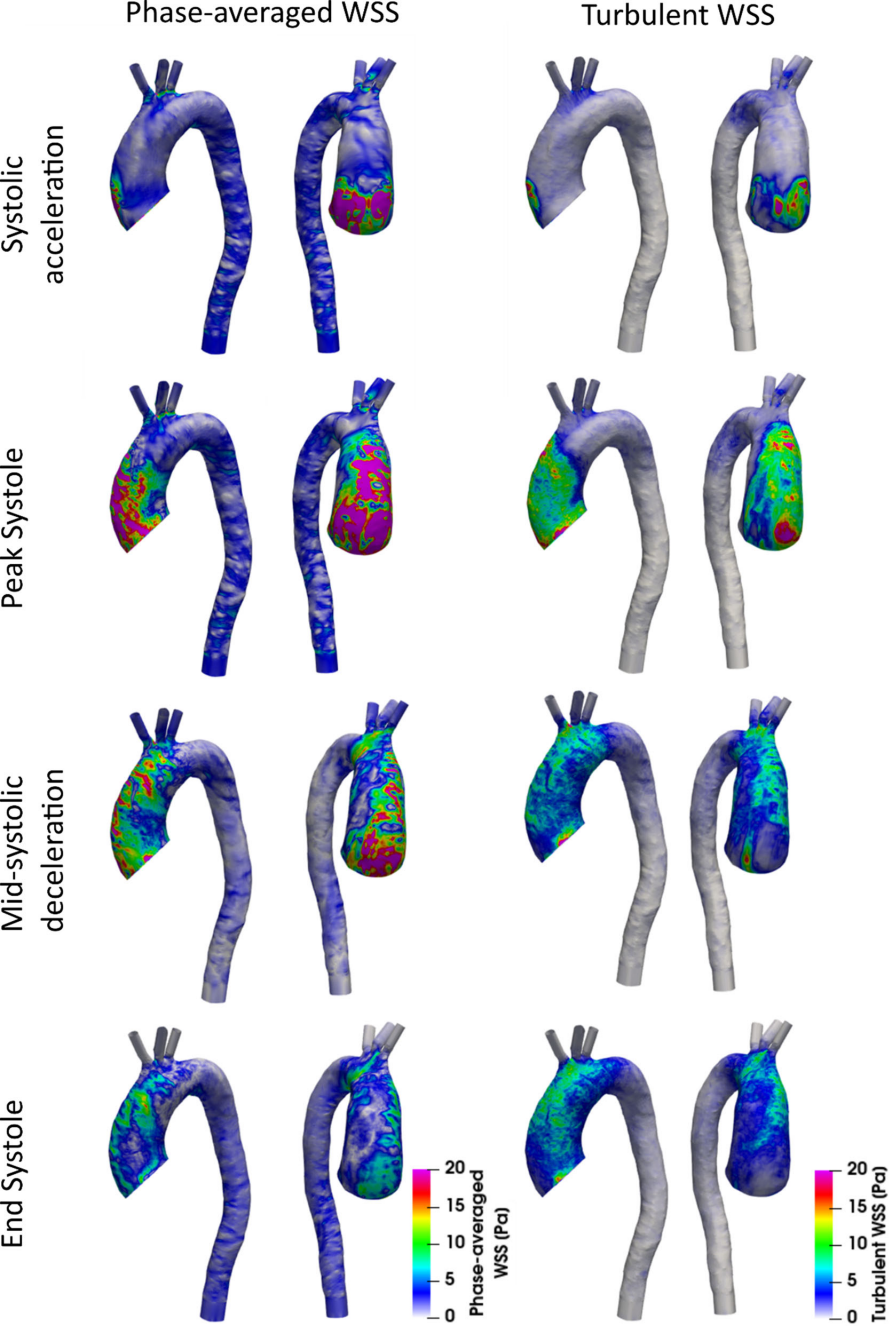
Surface contours of phase-averaged (laminar) and fluctuating (turbulent) wall shear stress at (top to bottom) systolic acceleration, peak systole, mid-systolic deceleration and end systole.

**Figure 8:**
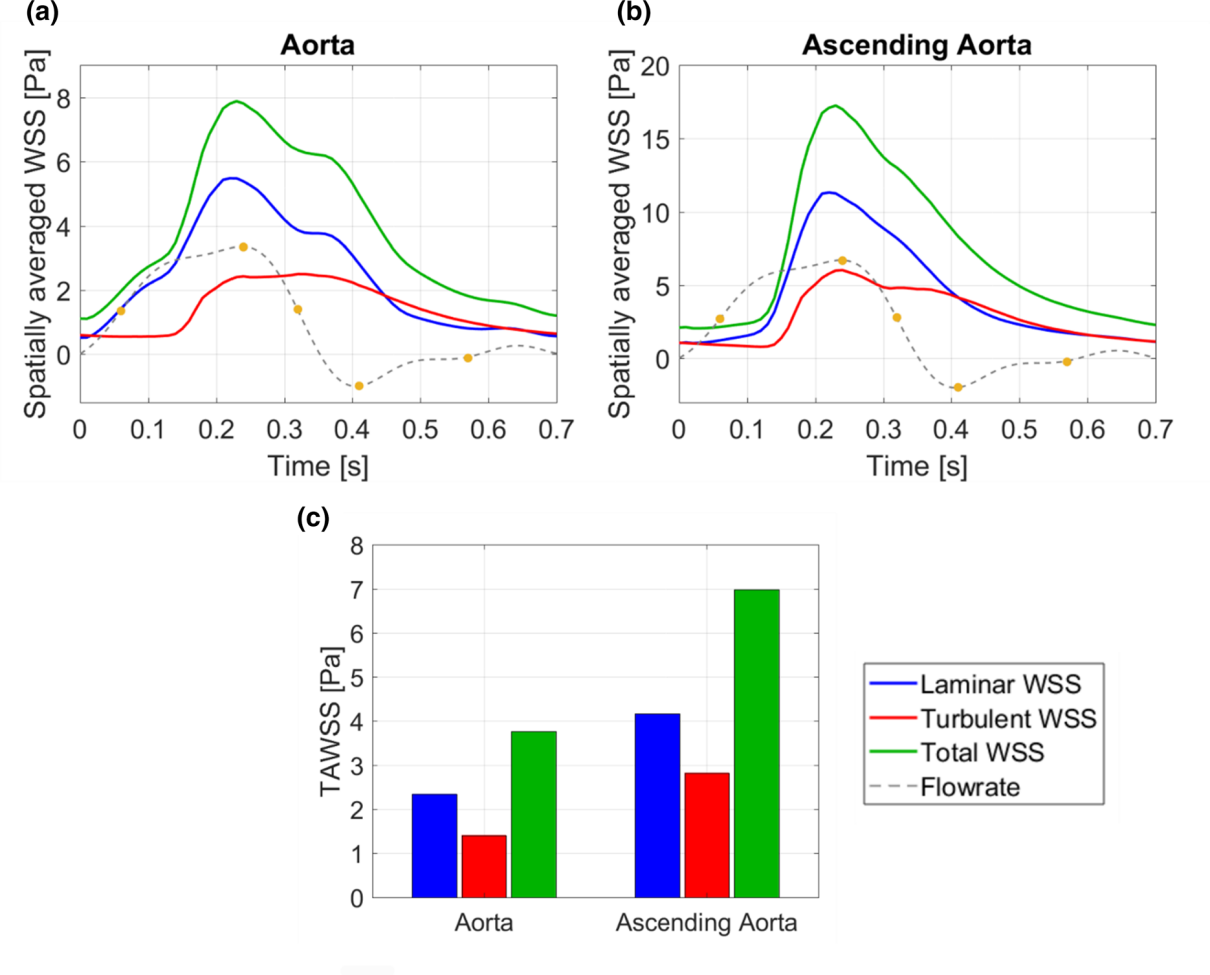
Wall shear stress components spatially averaged over (a) the entire aorta and (b) the ascending aorta plotted over a cardiac cycle. (c) Time-averaged wall shear stress spatially averaged over the entire aorta and ascending aorta. Plots show total wall shear stress alongside laminar WSS and turbulent WSS components. Key times throughout the cardiac cycle are highlighted and refer to maximum acceleration, peak systole, maximum deceleration, end systole and mid-diastole.

Surface contours of laminar and turbulent WSS at four key times throughout the cycle are shown in Fig. 7. Elevated values of turbulent WSS are present from late systolic acceleration through mid-diastole. In the ascending aorta, laminar and turbulent WSS are small during early systolic acceleration and increase rapidly in later systolic acceleration. Figure 8 shows the phase-averaged laminar, turbulent and total WSS over the cardiac cycle, spatially averaged over the entire aortic fluid domain (a) and averaged over the ascending aorta (b); where regions of highest WSS are present. Spatially averaged WSS components reach maximum values of 11 Pa (laminar) and 6 Pa (turbulent) in the ascending aorta both at peak systole, resulting in a total peak WSS of 17 Pa (Fig. 8a). At this point in the cardiac cycle, laminar and turbulent WSS start to exhibit different behaviours; laminar WSS steadily decreases during systolic deceleration whilst turbulent WSS shows a small decrease from the peak value but remains mostly constant. At end systole, laminar and turbulent WSS reach the same magnitude of 4 Pa. During diastole both stresses decrease gradually to ~ 1 Pa and turbulent WSS is marginally larger than laminar WSS. Similar trends are observed for WSS spatially averaged over the entire aorta (Fig. 8b), except for WSS magnitudes which are smaller owing to lower energy flows reaching the descending aorta as can be seen from the kinetic energies in Fig. 5.

Figure 8c shows the time-averaged wall shear stress (TAWSS) components spatially averaged over the entire aorta and the ascending aorta alone. Turbulent TAWSS account for 40% of the total TAWSS in the ascending aorta and 38% in the entire aorta. The total TAWSS spatially averaged over the entire aorta and ascending aorta is 3.8 and 7.0 Pa, respectively.

### Energy Loss

The energy required to maintain proper cardiac function, known as the stroke work, can be estimated from a patient’s stroke volume and mean arterial pressure.^41^ For patients with abnormal flow features, such as aortic valve stenosis, additional energy is needed to sustain cardiac function and this additional energy can be quantified using dissipation. Integrating the dissipation over a cardiac cycle gives the net energy loss per cardiac cycle. These energy losses are irreversible, meaning the energy cannot be recovered and is a direct measurement of the additional work required of the heart. Energy loss is a frictional loss and is the work done by a fluid on its adjacent layers due to shearing forces which are dissipated into heat. In disturbed flows, part of the total energy loss will be due to friction in the mean velocity field, known as viscous energy loss and part will be due to friction in the fluctuating velocity field, termed turbulent energy loss.

**Figure 9:**
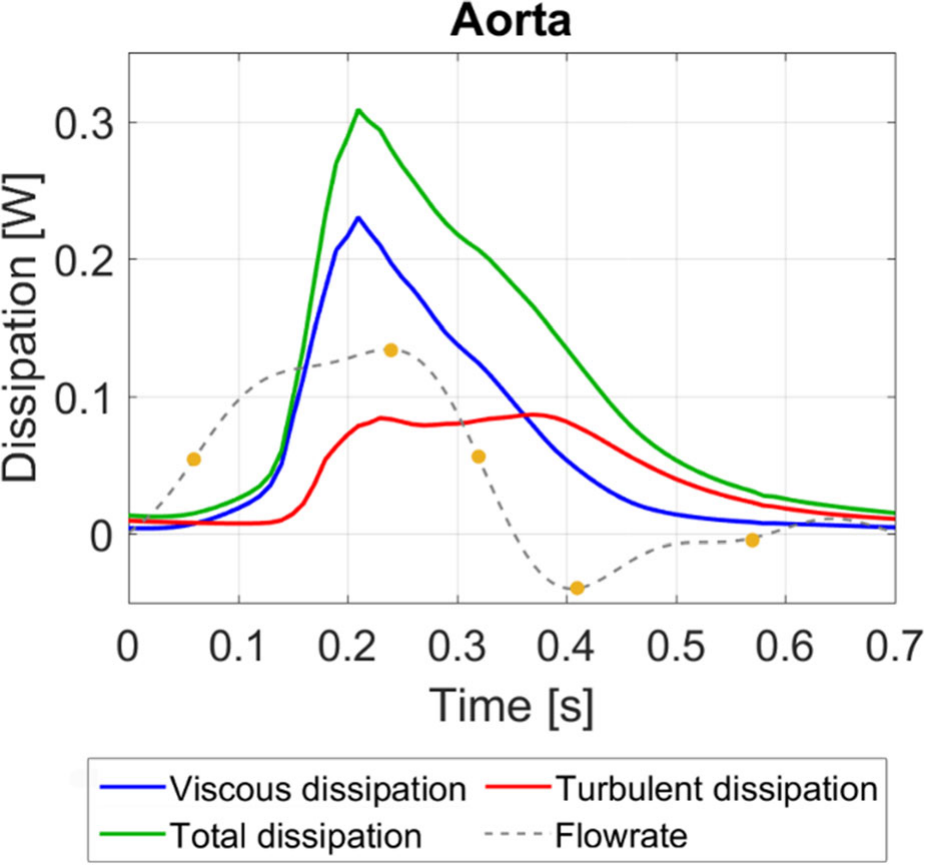
Dissipation (rate of energy loss) over the entire aorta. Plots show the viscous (laminar), turbulent and total dissipation. Key times throughout the cardiac cycle are highlighted.

This patient has an estimated stroke work of 1.12 Joules per cardiac cycle and this is calculated using the 4D flow MRI-derived inlet flowrate and the mean arterial pressure acquired from the same patient. Viscous and turbulence dissipation over a cardiac cycle account for irreversible net energy losses of 3.9% and 2.7% of the stroke work, respectively, with energy losses summarised in Table 2. Viscous, turbulent and total dissipation (Watts) phase-averaged over a cardiac cycle are plotted in Fig. 9 and it can be seen that the temporal distributions of viscous and turbulent dissipations are notably different. The viscous term increases rapidly during late systolic acceleration, peaking at 0.23 W just ahead of peak systole. Viscous dissipation decreases steadily at a slower rate during systolic deceleration and further decreases to almost zero (minimum 0.004 W) during diastole. Turbulent dissipation is small until late systolic acceleration, where it increases rapidly to 0.08 W at peak systole. Turbulence dissipation values remain mostly constant throughout the remainder of systolic deceleration, decreasing steadily throughout diastole to small amounts (minimum 0.007 W).

**Table 2:** Net energy loss over a cardiac cycle.

	Viscous	Turbulent	Total	Stroke work (J)
Net energy loss (J)	0.043	0.030	0.074	1.12
% of stroke work	3.9%	2.7%	6.6%	–

## Discussion

### Kinetic Energy

Mean kinetic energy is a product of primary flow features and as such, is high during systole when flow is ejected from the left ventricle into the ascending aorta. KE increases rapidly during systolic acceleration, reaching a maximum at peak systole and decreases from systolic deceleration to small values during diastole. Turbulent kinetic energy exhibits different behaviours throughout the cycle; TKE is small during early systole and increases rapidly at late systolic acceleration. TKE remains relatively constant throughout systolic deceleration and gradually decreases during diastole.

This patient does not have any pronounced geometrical features that may promote an increase in kinetic energy (e.g., aortic coarctation, sharp bend at the arch-descending aorta connection, unusual branch connections). As such this is a good example to simply assess the effects of aortic valve stenosis with a dilated ascending aorta—a common secondary disease in AVS patients. The primary source of turbulence production is the high velocity jet from the stenosed aortic valve entering the dilated ascending aorta, where velocities are low. This can be visualised in Fig. 4 by the high KE and low TKE in the jet core during systolic acceleration, but high levels of TKE in the surrounding shear regions (between jet and surrounding low velocity blood). This turbulence production is further amplified by two features; the dilated ascending aorta of this patient (an anatomical feature, providing space for turbulence to develop) and the highly skewed jet which impacts on the anterior vessel wall. Turbulence dissipates during diastole owing to the turbulent energy cascade; a lack of energy entering the system (aorta) and low kinetic energy in the mean flow are unable to sustain turbulence.

### Wall Shear Stress

During systolic acceleration when turbulence production is low, total WSS is dominated by the laminar component and turbulent WSS is small (Fig. 8). Both components of WSS increase rapidly during systolic acceleration when the high velocity jet from the stenosed valve impinges on the arterial wall. During systolic deceleration, laminar WSS decreases and turbulent WSS remains relatively constant owing to the additional time it takes for turbulence features to dissipate. Here, turbulence occupies the largest spatial region of the aorta and turbulent WSS values are relatively constant, accounting for ~ 40% of the total WSS in both the ascending and entire aorta. Laminar and turbulent WSS curves intersect in early diastole as mean kinetic energy rapidly decreases. During diastole, the kinetic energy in the system is small causing turbulence dissipation and consequently, turbulent WSS values steadily decrease. Due to low kinetic energy levels in the flow, laminar WSS also decreases at a higher rate during diastole and turbulent WSS exceeds mean WSS.

Regions of high turbulent WSS correspond with regions of high laminar WSS, with both occurring where high energy flow travels near the wall (Fig. 7). Throughout the cardiac cycle turbulent WSS is very small in the descending aorta (< 0.4 Pa), owing to the low levels of turbulence which dissipate ahead of the descending aorta. In the ascending aorta and arch, turbulent WSS are present throughout most of the cardiac cycle and are most significant from late systolic acceleration through mid-diastole.

It is well documented that endothelial cells play an important role in regulating the biological functions of the arterial wall. Exposure to high shear stresses for prolonged periods affects endothelial cell response, which promotes vascular remodelling and pathologies.^13^,^15^ Similarly, endothelial cell response is sensitive to space-time fluctuations of WSS that occur in disturbed flows. Fluctuating WSS is known to induce endothelial dysfunction,^11^,^14^ whereas high WSS in aortic valve disease is associated with extracellular matrix dysregulation and elastic fiber degeneration,^8^,^19^ which correlate with impaired tissue biomechanics. Clearly, aortic valve disease-related WSS parameters are an important biomarker for developing aortopathies. An exact threshold of WSS associated with developing aortopathies is not available however WSS values exceeding 3 Pa can be viewed as high^15^ and may promote vascular changes. In the ascending aorta, spatially-averaged laminar WSS reaches 11 Pa at peak systole and exceeds 3 Pa for 44% of the cardiac cycle (0.35 s). When considering both laminar and turbulent contributions, the peak total WSS is 17 Pa, and the 3 Pa threshold is exceeded for significantly longer; 68% of the cardiac cycle (0.54 s). It is evident that neglecting the turbulent contribution to WSS—even if the adopted numerical method accounts for turbulence—would significantly underpredict not only the total WSS but the length of time the arterial wall is subjected to these high shear stresses.

Time-averaged wall shear stresses (TAWSS) averaged over the entire aorta and ascending aorta are high with turbulent TAWSS accounting for 40% of the total TAWSS. With the inclusion of turbulent TAWSS, the total TAWSS now exceeds 3 Pa in both the entire aorta and ascending aorta with values of ~ 3.8 and 7.0 Pa. Understanding the severity of AVS by quantifying turbulence production may prove a useful biomarker for understanding the onset and progression of secondary diseases that are associated with aortic valve diseases.

### Energy Loss

There have been studies which estimate either viscous^5^,^18^,^27^ or turbulent^20^ energy losses from 4D flow MRI data or numerical simulation in aortic and idealised flows. Gilmanov *et al.*^18^ conducted patient-specific fluid-structure interaction simulations of calcified aortic valves. In the valve with a high degree of calcification; which obstructs the effective valve orifice area producing aortic flows similar to AVS, a peak viscous dissipation of ~ 0.17 W was observed which is of similar magnitude to the peak viscous dissipation in this study (0.23 W). Yap *et al.*^47^ performed experiments of a healthy porcine aortic valve connected to a simplified aorta model. Experiments were performed with and without artificially induced valve stenosis, over a range of stroke volumes and heart rates. They estimated total dissipation and total energy loss across the valve using a simplified energy loss equation derived from the Navier-Stokes equations. At similar stroke volumes and heart rates to the current study, they observed net energy losses on the order of ~ 0.09 J and ~ 0.2 to 0.3 J for their mild and moderate stenosis cases, respectively. Our values for net energy loss and viscous dissipation are of similar magnitudes to those reported in literature,^18^,^47^ despite differences in methods. Previous studies have estimated either the viscous, turbulent or total dissipation and/or energy losses in aortic flows, but none have quantified the individual contributions from viscous and turbulent dissipation—a key step in understanding not only the net effect of aortic valve disease on energy loss but also the isolated effects of turbulence on energy loss.

In this study, total irreversible energy losses account for 6.6% of the stroke work, with turbulent energy losses accounting for 41% of the total energy loss. The majority of viscous energy losses throughout the aorta originate from the boundary layer and other contributions are from the high shear layers between the stenosed jet and surrounding low velocity blood in the ascending aorta, as well as the shear layers where the stenosed jet impinges on the anterior vessel wall. Turbulent energy losses originate wherever there is turbulence (see TKE renderings, Fig. 5). Even though peak values of turbulence dissipation (0.08 W) are smaller than viscous dissipation (0.23 W), turbulence dissipation exceeds viscous dissipation values for 59% of the cardiac cycle (late systolic deceleration through diastole and early systolic acceleration). This is because turbulent features, albeit small, are present in the ascending aorta and arch throughout the cardiac cycle.

### Study Limitations

In the present study the aortic wall was assumed to be rigid and blood was assumed to be Newtonian. A compliant aortic wall may affect turbulence intensities and rates of dissipation, particularly throughout diastole when the energy accumulated in the arterial wall during systole is released. Neglecting wall deformation can overestimate WSS in laminar arterial flows^9^ and in transitional flow in a thoracic aortic aneurysm^38^; the latter also found higher turbulence intensity in the fluid-structure interaction (FSI) model compared to the rigid model. Nevertheless, the patient included in the present study had a significantly dilated ascending aorta (~ 5 cm diameter) and aneurysmal walls are known to be stiffer than a typical healthy aorta. As demonstrated in a recent study of 11 ascending aortic aneurysms, rigid wall CFD and 2-way FSI simulations produced comparable WSS predictions.^26^ Therefore, it would be reasonable to expect the effect of wall deformation on the predicted WSS to be limited in our case. However, further studies are still needed to assess the influence of wall compliance on other turbulence related parameters. While the assumption of a constant Newtonian viscosity for blood flow in the aorta is generally acceptable, it has been shown that the non-Newtonian rheology of blood could reduce turbulence levels.^4^

It is also worth noting that uncertainties originating from MRI data acquisition and processing can propagate and introduce uncertainties in the simulated output. A numerical study of the healthy aorta conducted by Bozzi *et al*.^10^ found uncertainties up to 30% in the calculated WSS. Further work would be required to estimate uncertainty propagation associated with the variables in the current study. All efforts have been taken to minimise uncertainty and error in terms of the choice of numerical procedure and boundary conditions. This is also mitigated through careful mesh and time-step sensitivity studies, as well as detailed comparisons with *in vivo* measurements with 4D flow MRI.

Finally, this study was conducted on a single patient; therefore, more AVS cases would need to be included before the findings can be generalised. Nonetheless, the methodology presented in this study is readily applicable to not only AVS but also cases of other aortic valve diseases.

## Conclusions

In this study we analysed flow in a patient-specific aorta with aortic valve stenosis and dilated ascending aorta. Comparisons of LES predicted and *in vivo* MRI measured velocities demonstrated good qualitative and quantitative agreement. Our analysis of kinetic energy measures showed that turbulence was present primarily in the ascending aorta and aortic arch from late systolic acceleration through end systole. The main causes of turbulence production in this patient were: (i) the high velocity jet, (ii) severe skewness of the jet which impacted on the arterial wall and (iii) a dilated ascending aorta providing sufficient space for turbulence to develop.

Our quantitative analysis of the impact of turbulence on wall shear stress revealed that turbulent WSS accounted for 40% of the total WSS in the ascending aorta and 38% in the entire aorta. Neglecting the turbulent contribution to WSS would result in a significant underestimation of the total WSS in this case. Furthermore, total WSS in the ascending aorta exceeded 3Pa (a threshold for potential vascular changes) for 68% of the cardiac cycle. This period of exposure would be significantly less (65% underprediction) if turbulent WSS was not included. The results from this study suggest that inclusion of turbulent WSS in disturbed aortic flows may aid in our attempts to better understand the relationship between WSS-related parameters and aortic wall disease progression, although more cases would be needed. Viscous and turbulent irreversible energy losses were calculated using phase-averaged and turbulent velocity gradient fields and accounted for 3.9 and 2.7% of the total stroke work, respectively. In this study, for the first time, we have quantified aortic valve stenosis turbulence production against left ventricular load in a single patient-specific computational model.

## Supplementary Information

Below is the link to the electronic supplementary material.

Supplementary material 1 (DOCX 516 kb)

## Abbreviations

AVS: Aortic valve stenosis
LES: Large-eddy simulation
MRI: Magnetic resonance imaging
WSS: Wall shear stress
TAWSS: Time-averaged wall shear stress
TKE: Turbulence kinetic energy
KE: Mean kinetic energy
RMS: Root-mean-square
