# Supplementary material for: Analysis of Turbulence Effects in a Patient-Specific Aorta with Aortic Valve Stenosis
Source: Cardiovasc Eng Technol. 2021 Apr 7;12(4):438–53. doi: 10.1007/s13239-021-00536-9 (PMC8354935; doi:10.1007/s13239-021-00536-9)
Supplement: Supplementary file 1 — Supplementary material 1 (DOCX 516 kb) [file 13239_2021_536_MOESM1_ESM.docx]

# Supplementary Material

## Supplementary Material S1. Windkessel Parameters

Table S1: Values for parameters used in the three-element Windkessel model. $R_{1}$= proximal resistance, $R_{2}$= distal resistance, $C$= arterial compliance. BCA = brachiocephalic artery, LCCA = left common carotid artery, LSA = left subclavian artery and DAo = descending aorta.

|  | $\boldsymbol{R}_{\boldsymbol{1}}$  $\boldsymbol{[}\boldsymbol{10}^{\boldsymbol{7}}\boldsymbol{Pa s}\boldsymbol{m}^{\boldsymbol{-3}}\boldsymbol{]}$ | $\boldsymbol{R}_{\boldsymbol{2}}$  $\boldsymbol{[}\boldsymbol{10}^{\boldsymbol{8}}\boldsymbol{Pa s}\boldsymbol{m}^{\boldsymbol{-3}}\boldsymbol{]}$ | $\boldsymbol{C}$  $\boldsymbol{[}\boldsymbol{10}^{\boldsymbol{-10}} \boldsymbol{m}^{\boldsymbol{3}}\boldsymbol{P}\boldsymbol{a}^{\boldsymbol{-1}}\boldsymbol{]}$ |
| --- | --- | --- | --- |
| BCA | 12.1 | 30.6 | 6.0 |
| LCCA | 9.1 | 24.1 | 7.7 |
| LSA | 9.8 | 25.7 | 7.2 |
| DAo | 1.4 | 2.0 | 88.1 |

## Supplementary Material S2. Computational Mesh

Table S2. Characteristics of the three meshes.

| Mesh characteristics | | | | Percentage error, relative to M3 | | |  |
| --- | --- | --- | --- | --- | --- | --- | --- |
| Integrated Region | Mesh name | Number of cells [millions] | Mean cell length [mm] | Mean WSS  [%] | Turbulent WSS  [%] | TKE  [%] | Contribution of SGS model [%] |
| Entire Aorta  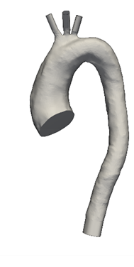 | M1 | 3.4 | 0.79 | 10.6 | 4.6 | 1.0 | 6.0 |
|  | M2 | 7.4 | 0.53 | 0.2 | 2.3 | 0.2 | 4.0 |
|  | M3 | 14.3 | 0.43 | - | - | - | 2.9 |
| Ascending Aorta  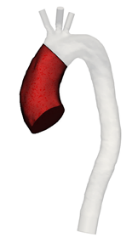 | M1 |  |  | 7.5 | 8.8 | 6.3 | 7.5 |
|  | M2 |  |  | 0.1 | 2.2 | 1.9 | 4.9 |
|  | M3 |  |  | - | - | - | 3.7 |
| Here WSS and TKE are the wall shear stress and turbulence kinetic energy. The final column is the subgrid-scale model contribution to the simulation and represents the percentage of the flow field that is modelled. | | | | | | | |

## Supplementary Material S3. Qualitative comparisons

Figure 1 shows three components of velocity contours at three slice locations along the aorta, at three time points corresponding to systolic acceleration, peak systole and systolic deceleration. All velocity component contours extracted from 4D flow MRI and LES simulations showed good agreement at each slice location, across all times, demonstrating good qualitative agreement.


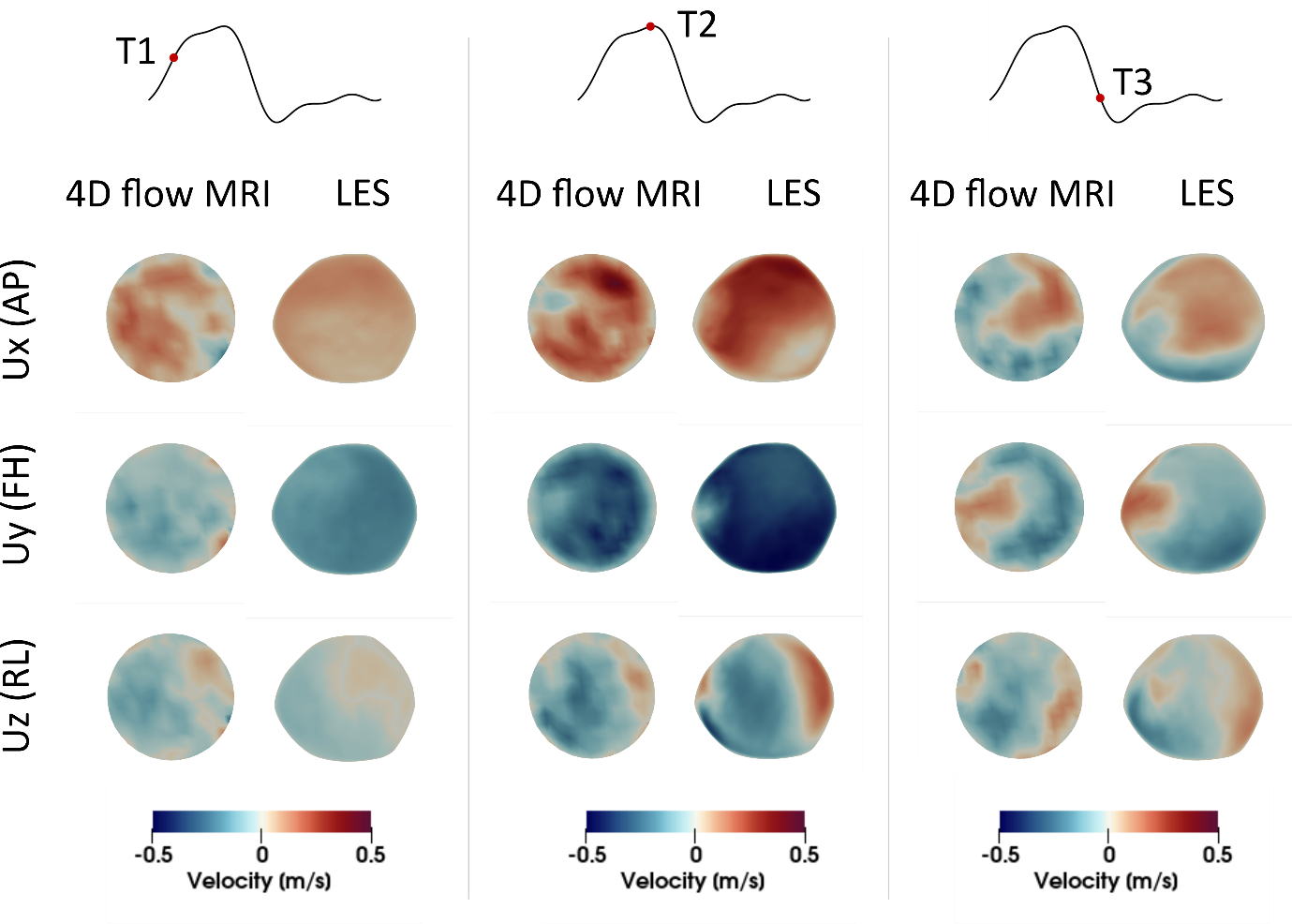


Figure 1. Comparison of velocity contours between 4D flow MRI and LES results. Velocity contours shown are at a plane in the descending aorta, plotted over three time points corresponding to systolic acceleration, peak systole and systolic deceleration. The three components of velocity are anterior-posterior (AP), foot-head (FH) and right-left (RL) which correspond to x, y, z co-ordinates respectively.
